# A Monstrosity

**Published:** 1885-10

**Authors:** T. M. Holmes

**Affiliations:** Rome, Ga.


### A MONSTROSITY.

BY T. M. HOLMES, M. D., ROME, GA.

On January 4th, of this year, I was hurriedly summoned to attend a case of labor several miles in the country. The messenger, the husband of the woman, informed me that his wife had been in labor for several hours, and that the head of the child was born, and that something was wrong, and he feared his wife would die.

On my arrival I found matters very much as he had represented. The woman was suffering a great deal. On examination, I found the child to be dead, and found almost complete uterine inertia. This I thought to be a sufficient reason for the delay of delivery, and hoped that ergot, manipulation and traction would soon accomplish the desired end. Hence, I proceeded at once to put into execution these measures, but imagine my amazement when my whole strength was brought to bear without making the least progress. I then resorted to a bandage around the neck and waist, which resulted in drawing one arm almost out of its socket, and in quite beheading the child. On starting from my office I had failed to carry my forceps, so that I was now thrown upon my wits to devise some means of handling this monster. I conceived the idea of substituting, instead of the ordinary hooks, a pair of old-fashioned pot hooks. This was an adaptation of very humble means for the accomplishing of a much desired end, but it worked like a charm. The hooks were placed in each side below the arm-pits, and for the second time within a month, and the only times in my life, I was reduced to the necessity of calling upon the husband to unite his strength with mine in the delivery of his wife. The former case, like this one, was characterized by an unusually abnormal condition of things. Its sequelæ and results of treatment I hope to be able to report in the future. In this case delivery was soon accomplished to the satisfaction and delight of all concerned. The *modus operandi* was perhaps a little unique, but none the less effective, for success was the reward of our labor.

The shape of this fœtus was most remarkable, being that of a balloon, the fundus being at the breech. This was the secret of the whole trouble. I could now see why the difficulty of delivery. Its weight was twelve and a half pounds. On opening the abdomen three pounds of an opaque sanguinolent fluid ran out. The intestines were intact, but there was no stomach nor spleen, and the lungs and kidneys were the merest rudiments.

The interesting feature in the case was the formidable delivery, when, so far as could be discovered, there was no cause for it, and which cause, when known, proved to be so unusual. The mother made a good recovery.

